# Poly[[(methanol)(μ_4_-2,4,5,6-tetra­fluoro­benzene-1,3-dicarboxyl­ato)copper(II)] methanol monosolvate]

**DOI:** 10.1107/S1600536812020740

**Published:** 2012-05-16

**Authors:** Dan Yan, Qian Duan

**Affiliations:** a School of Materials Science and Engineering, Changchun University of Science and Technology, Changchun 130022, People’s Republic of China

## Abstract

In the title compound, {[Cu(C_8_F_4_O_4_)(CH_3_OH)]·CH_3_OH}_*n*_, two Cu^II^ atoms are bridged by four carboxyl­ate groups, forming the well known paddle-wheel secondary building unit (SBU) with axial methanol ligands. In each ligand, the dihedral angles between the benzene ring and the two carboxyl­ate groups are 80.43 (17) and 62.5 (4)°. Within each SBU, the four carboxyl­ate groups come from four symmetry-equivalent tetra­fluoro­isophthalate ligands. Each tetra­fluoro­isophthalate group connects two SBUs, forming a layered structure . In the crystal, O—H⋯O hydrogen bonds involving the free and ligated methanol mol­ecules link the mol­ecules into a three-dimensional supra­molecular network.

## Related literature

For background to coordination polymers, see: Kim *et al.* (2001 ▶); Kitagawa *et al.* (2004 ▶). For applications of coordination polymers, see: Wang *et al.* (2009 ▶); Dincă & Long (2008 ▶); Furukawa *et al.* (2008 ▶). For information on fluorinated coordination polymers, see: Yang *et al.* (2007 ▶); Hulvey *et al.* (2009 ▶).
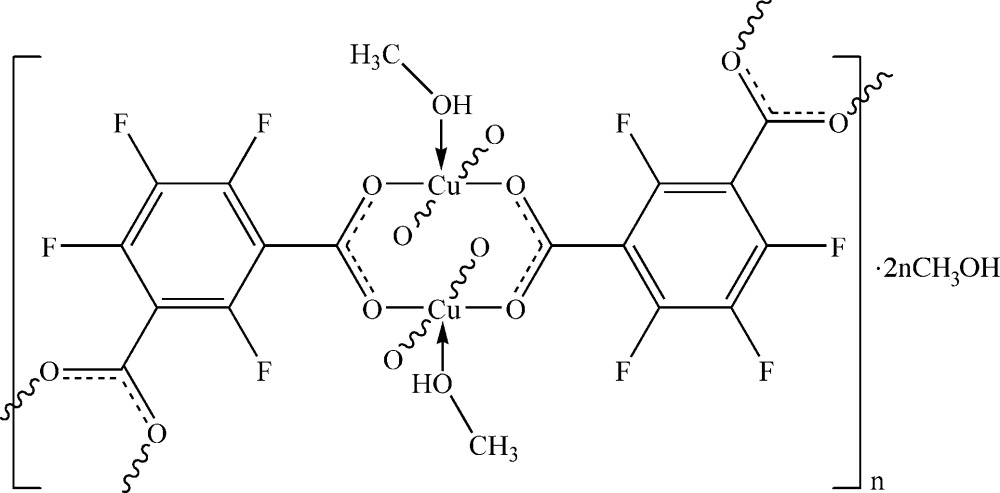


## Experimental

### 

#### Crystal data

[Cu(C_8_F_4_O_4_)(CH_4_O)]·CH_4_O
*M* *_r_* = 363.70
Monoclinic, 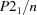

*a* = 8.6542 (7) Å
*b* = 12.1882 (10) Å
*c* = 12.4272 (10) Å
β = 98.390 (1)°
*V* = 1296.78 (18) Å^3^
*Z* = 4
Mo *K*α radiation
μ = 1.76 mm^−1^
*T* = 200 K
0.34 × 0.22 × 0.19 mm

#### Data collection

Bruker APEXII CCD area-detector diffractometer
Absorption correction: multi-scan (*SADABS*; Sheldrick, 2003 ▶) *T*_min_ = 0.621, *T*_max_ = 0.715
8122 measured reflections
2575 independent reflections
2365 reflections with *I* > 2σ(*I*)
*R*_int_ = 0.017

#### Refinement

*R*[*F* ^2^ > 2σ(*F* ^2^)] = 0.033
*wR*(*F* ^2^) = 0.092
*S* = 1.07
2575 reflections
196 parameters
1 restraint
H atoms treated by a mixture of independent and constrained refinement
Δρ_max_ = 1.05 e Å^−3^
Δρ_min_ = −0.40 e Å^−3^

### 

Data collection: *APEX2* (Bruker, 2007 ▶); cell refinement: *SAINT* (Bruker, 2007 ▶); data reduction: *SAINT*; program(s) used to solve structure: *SHELXS97* (Sheldrick, 2008 ▶); program(s) used to refine structure: *SHELXL97* (Sheldrick, 2008 ▶); molecular graphics: *XP* in *SHELXTL* (Sheldrick, 2008 ▶); software used to prepare material for publication: *SHELXL97*.

## Supplementary Material

Crystal structure: contains datablock(s) I, global. DOI: 10.1107/S1600536812020740/pk2400sup1.cif

Structure factors: contains datablock(s) I. DOI: 10.1107/S1600536812020740/pk2400Isup2.hkl

Additional supplementary materials:  crystallographic information; 3D view; checkCIF report

## Figures and Tables

**Table 1:** Selected bond lengths (Å)

Cu1—O2^i^	1.9600 (18)
Cu1—O1	1.9650 (18)
Cu1—O3^ii^	1.9656 (18)
Cu1—O4^iii^	1.9734 (17)
Cu1—O5	2.0834 (19)

Symmetry codes: (i) 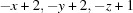; (ii) 
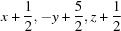
; (iii) 
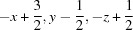
.

**Table 2:** Hydrogen-bond geometry (Å, °)

*D*—H⋯*A*	*D*—H	H⋯*A*	*D*⋯*A*	*D*—H⋯*A*
O5—H5⋯O6^iv^	0.84 (1)	1.80 (1)	2.637 (3)	173 (4)
O6—H6⋯O4^v^	0.82	2.02	2.828 (3)	169

Symmetry codes: (iv) 
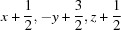
; (v) 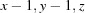.

**Fig. 1.**
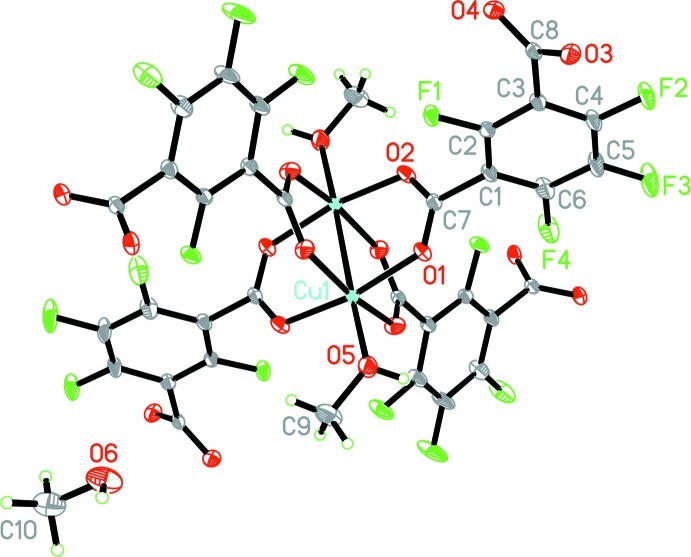
An ellipsoid plot (30% probability level) of the paddlewheel SBU showing the labelled asymmetric unit. Hydrogen atoms are drawn as small arbitrary spheres.

**Fig. 2.**
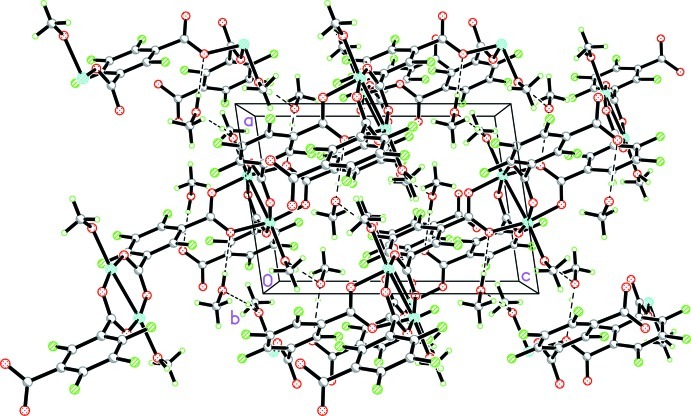
A view of the two-dimensional packing of the title compound, the hydrogen bonding interactions are shown as broken lines.

## supplementary crystallographic information

### Comment

The design and synthesis of coordination polymers is an active area of research as these compounds have potential uses in gas storage, catalysis, magnetism and so on. The Omary and Cheetham groups have both reported interesting hydrogen adsorption properties in porous coordination polymers containing fluorinated ligands (Yang *et al.* 2007; Hulvey *et al.* 2009). Indeed, most of the reports to date of coordination polymers containing perfluorinated dicarboxylates involve a second ligand, which is typically a simple, nonfluorinated, nitrogen-containing molecule. The well known paddlewheel secondary building unit (*M*_2_(O_2_CR)_4_*L*_2_, *M*=Cu, Zn, *etc*.; *L*=terminal ligand) has been used extensively in generating porous coordination polymers. Here, we report a perfluorinated coordination polymer (I), {[Cu(C_8_F_4_O_4_)(CH_3_OH)].CH_3_OH}_n_, which is constructed using the paddlewheel SBU Cu_2_(O_2_CR)_4_*L*_2_ (*L*=CH_3_OH).

The asymmetric unit is composed of one Cu^II^ center, one tetrafluoroisophthalate anion, one coordinated methanol ligand, and one methanol solvent molecule (Fig. 1). Each Cu^II^ ion is five-coordinated by four oxygen donors from four different tetrafluoroisophthalate ligands and one oxygen atom from a terminal methanol molecule. In the paddlewheel SBU, the two copper ions are separated by 2.6622 (6) Å. Each SBU connects four tetrafluoroisophthalate ligands, and each tetrafluoroisophthalate group connect two SBUs to form a two dimensional layered structure (Fig. 2). Adjacent parallel layers are connected by O—H···O hydrogen bonds between guest methanol molecules and the coordinated methanol molecules to create a three-dimensional supramolecular network.

### Experimental

Compound I was obtained by layering 5 ml of a methanol solution containing 2,4,5,6-tetrafluoro-1,3-benzenedicarboxylic acid (23 mg, 0.10 mmol) and 2,6-lutidine (0.034 ml, 0.30 mmol) onto 5 ml of a methanol/nitrobenzene solution (1.5:1, *v*/*v*) containing Cu(NO_3_)_2_.2.5H_2_O (23 mg, 0.10 mmol). Green crystals formed at the interlayer boundary within one week. After two weeks, blue block-shaped crystals of the title compound suitable for X-ray diffraction were obtained by slow diffusion of the solvents in 26% yield (9.5 mg, based on the ligand).

### Refinement

All H atoms bound to C atoms and O—H hydrogen atoms of the free methanol molecules were assigned to calculated positions with C—H = 0.96 Å, O—H = 0.82 Å, and refined using a riding model, with *U*_iso_(H)=1.5 *U*_eq_(C,O). O—H hydrogen atoms of the coordinated methanol molecules were found in difference Fourier maps and refined isotropically with the distance restraint: O—H = 0.85 Å and *U*_iso_(H) = 1.5 *U*_eq_(O).

### Crystal data

**Table:**

[Cu(C_8_F_4_O_4_)(CH_4_O)]·CH_4_O	*F*(000) = 724
*M**_r_* = 363.70	*D*_x_ = 1.863 Mg m^−^^3^
Monoclinic, *P*2_1_/*n*	Mo *K*α radiation, λ = 0.71073 Å
Hall symbol: -P 2yn	Cell parameters from 2575 reflections
*a* = 8.6542 (7) Å	θ = 2.4–26.1°
*b* = 12.1882 (10) Å	µ = 1.76 mm^−^^1^
*c* = 12.4272 (10) Å	*T* = 200 K
β = 98.390 (1)°	Block, green
*V* = 1296.78 (18) Å^3^	0.34 × 0.22 × 0.19 mm
*Z* = 4	

### Data collection

**Table:**

Bruker APEXII CCD area-detector diffractometer	2575 independent reflections
Radiation source: fine-focus sealed tube	2365 reflections with *I* > 2σ(*I*)
Graphite monochromator	*R*_int_ = 0.017
Detector resolution: 9.00 pixels mm^-1^	θ_max_ = 26.1°, θ_min_ = 2.4°
φ and ω scans	*h* = −10→10
Absorption correction: multi-scan (*SADABS*; Sheldrick, 2003)	*k* = −15→14
*T*_min_ = 0.621, *T*_max_ = 0.715	*l* = −13→15
8122 measured reflections	

### Refinement

**Table:**

Refinement on *F*^2^	Primary atom site location: structure-invariant direct methods
Least-squares matrix: full	Secondary atom site location: difference Fourier map
*R*[*F*^2^ > 2σ(*F*^2^)] = 0.033	Hydrogen site location: inferred from neighbouring sites
*wR*(*F*^2^) = 0.092	H atoms treated by a mixture of independent and constrained refinement
*S* = 1.07	*w* = 1/[σ^2^(*F*_o_^2^) + (0.0551*P*)^2^ + 1.2274*P*] where *P* = (*F*_o_^2^ + 2*F*_c_^2^)/3
2575 reflections	(Δ/σ)_max_ = 0.001
196 parameters	Δρ_max_ = 1.05 e Å^−^^3^
1 restraint	Δρ_min_ = −0.40 e Å^−^^3^

### Special details

**Table:**

Geometry. All e.s.d.'s (except the e.s.d. in the dihedral angle between two l.s. planes) are estimated using the full covariance matrix. The cell e.s.d.'s are taken into account individually in the estimation of e.s.d.'s in distances, angles and torsion angles; correlations between e.s.d.'s in cell parameters are only used when they are defined by crystal symmetry. An approximate (isotropic) treatment of cell e.s.d.'s is used for estimating e.s.d.'s involving l.s. planes.
Refinement. Refinement of *F*^2^ against ALL reflections. The weighted *R*-factor *wR* and goodness of fit *S* are based on *F*^2^, conventional *R*-factors *R* are based on *F*, with *F* set to zero for negative *F*^2^. The threshold expression of *F*^2^ > σ(*F*^2^) is used only for calculating *R*-factors(gt) *etc*. and is not relevant to the choice of reflections for refinement. *R*-factors based on *F*^2^ are statistically about twice as large as those based on *F*, and *R*- factors based on ALL data will be even larger.

### Fractional atomic coordinates and isotropic or equivalent isotropic displacement parameters (Å^2^)

**Table:**

	*x*	*y*	*z*	*U*_iso_*/*U*_eq_	
Cu1	0.86874 (3)	0.96498 (2)	0.53265 (2)	0.01964 (12)	
C1	0.8110 (3)	1.2979 (2)	0.4412 (2)	0.0263 (5)	
C2	0.7454 (3)	1.32560 (19)	0.33704 (19)	0.0244 (5)	
C3	0.6682 (3)	1.4235 (2)	0.30967 (19)	0.0253 (5)	
C4	0.6579 (4)	1.4958 (2)	0.3942 (2)	0.0364 (7)	
C5	0.7222 (5)	1.4715 (2)	0.4996 (2)	0.0471 (9)	
C6	0.7980 (4)	1.3732 (2)	0.5220 (2)	0.0412 (7)	
C7	0.8867 (3)	1.18712 (19)	0.46554 (18)	0.0237 (5)	
C8	0.6012 (3)	1.45109 (18)	0.1942 (2)	0.0239 (5)	
C9	0.6040 (4)	0.8097 (3)	0.5897 (3)	0.0528 (9)	
H9A	0.5000	0.8111	0.6079	0.079*	
H9B	0.6736	0.7776	0.6485	0.079*	
H9C	0.6053	0.7668	0.5250	0.079*	
C10	0.0958 (4)	0.3426 (3)	0.2112 (3)	0.0545 (9)	
H10A	0.2061	0.3549	0.2157	0.082*	
H10B	0.0624	0.2914	0.1538	0.082*	
H10C	0.0735	0.3132	0.2790	0.082*	
F1	0.7529 (2)	1.25055 (12)	0.25928 (11)	0.0331 (4)	
F2	0.5866 (3)	1.59239 (14)	0.37550 (13)	0.0523 (5)	
F3	0.7098 (4)	1.54384 (16)	0.57961 (16)	0.0796 (9)	
F4	0.8600 (3)	1.35034 (16)	0.62475 (14)	0.0636 (6)	
O1	0.8026 (2)	1.11737 (14)	0.50171 (15)	0.0307 (4)	
O2	1.0228 (2)	1.17630 (14)	0.44677 (15)	0.0313 (4)	
O3	0.4671 (2)	1.48965 (17)	0.17844 (14)	0.0319 (4)	
O4	0.6883 (2)	1.43243 (16)	0.12365 (13)	0.0285 (4)	
O5	0.6524 (2)	0.91720 (17)	0.57154 (19)	0.0400 (5)	
H5	0.601 (4)	0.960 (2)	0.606 (3)	0.057 (12)*	
O6	0.0148 (3)	0.4435 (2)	0.1894 (2)	0.0569 (7)	
H6	−0.0781	0.4313	0.1697	0.085*	

### Atomic displacement parameters (Å^2^)

**Table:**

	*U*^11^	*U*^22^	*U*^33^	*U*^12^	*U*^13^	*U*^23^
Cu1	0.02543 (18)	0.01590 (18)	0.01669 (18)	−0.00318 (10)	0.00001 (11)	0.00040 (10)
C1	0.0362 (13)	0.0189 (11)	0.0219 (12)	0.0017 (10)	−0.0022 (10)	0.0017 (9)
C2	0.0350 (13)	0.0201 (11)	0.0173 (11)	0.0010 (10)	0.0014 (9)	−0.0004 (9)
C3	0.0351 (13)	0.0206 (11)	0.0183 (11)	0.0019 (10)	−0.0023 (9)	0.0010 (9)
C4	0.0620 (19)	0.0204 (13)	0.0239 (13)	0.0141 (13)	−0.0028 (12)	0.0015 (10)
C5	0.088 (3)	0.0274 (15)	0.0208 (14)	0.0171 (14)	−0.0073 (15)	−0.0089 (10)
C6	0.073 (2)	0.0284 (14)	0.0171 (12)	0.0104 (14)	−0.0107 (12)	0.0024 (11)
C7	0.0353 (13)	0.0181 (11)	0.0155 (11)	0.0014 (9)	−0.0037 (9)	0.0005 (9)
C8	0.0335 (13)	0.0162 (11)	0.0200 (12)	0.0003 (9)	−0.0022 (10)	−0.0006 (9)
C9	0.0485 (19)	0.0407 (18)	0.070 (2)	−0.0086 (14)	0.0117 (17)	0.0131 (16)
C10	0.0519 (19)	0.061 (2)	0.049 (2)	−0.0095 (17)	0.0024 (15)	0.0140 (17)
F1	0.0550 (10)	0.0226 (7)	0.0197 (7)	0.0083 (6)	−0.0017 (6)	−0.0035 (6)
F2	0.0966 (15)	0.0268 (8)	0.0287 (9)	0.0282 (9)	−0.0068 (9)	−0.0013 (7)
F3	0.166 (3)	0.0405 (12)	0.0247 (10)	0.0440 (13)	−0.0131 (12)	−0.0126 (8)
F4	0.1242 (19)	0.0373 (10)	0.0196 (8)	0.0251 (11)	−0.0217 (9)	−0.0025 (7)
O1	0.0374 (10)	0.0191 (8)	0.0356 (10)	0.0020 (7)	0.0055 (8)	0.0043 (7)
O2	0.0369 (10)	0.0207 (8)	0.0359 (10)	0.0013 (7)	0.0046 (8)	0.0085 (7)
O3	0.0376 (10)	0.0376 (10)	0.0192 (9)	0.0111 (8)	0.0001 (7)	0.0032 (8)
O4	0.0319 (9)	0.0330 (9)	0.0190 (8)	0.0050 (8)	−0.0016 (7)	0.0054 (7)
O5	0.0375 (11)	0.0297 (10)	0.0566 (13)	−0.0091 (8)	0.0192 (10)	−0.0092 (9)
O6	0.0374 (12)	0.0643 (15)	0.0666 (17)	−0.0080 (11)	−0.0002 (11)	0.0208 (13)

### Geometric parameters (Å, º)

**Table:**

Cu1—O2^i^	1.9600 (18)	C7—O2	1.241 (3)
Cu1—O1	1.9650 (18)	C7—O1	1.245 (3)
Cu1—O3^ii^	1.9656 (18)	C8—O3	1.240 (3)
Cu1—O4^iii^	1.9734 (17)	C8—O4	1.258 (3)
Cu1—O5	2.0834 (19)	C9—O5	1.404 (4)
Cu1—Cu1^i^	2.6622 (6)	C9—H9A	0.9600
C1—C6	1.377 (4)	C9—H9B	0.9600
C1—C2	1.378 (3)	C9—H9C	0.9600
C1—C7	1.512 (3)	C10—O6	1.422 (5)
C2—F1	1.339 (3)	C10—H10A	0.9600
C2—C3	1.385 (3)	C10—H10B	0.9600
C3—C4	1.384 (4)	C10—H10C	0.9600
C3—C8	1.505 (3)	O2—Cu1^i^	1.9600 (18)
C4—F2	1.334 (3)	O3—Cu1^iv^	1.9656 (18)
C4—C5	1.379 (4)	O4—Cu1^v^	1.9733 (17)
C5—F3	1.344 (3)	O5—H5	0.842 (10)
C5—C6	1.375 (4)	O6—H6	0.8200
C6—F4	1.340 (3)		
			
O2^i^—Cu1—O1	167.50 (8)	F4—C6—C5	119.1 (3)
O2^i^—Cu1—O3^ii^	89.55 (8)	F4—C6—C1	119.5 (2)
O1—Cu1—O3^ii^	89.38 (8)	C5—C6—C1	121.4 (2)
O2^i^—Cu1—O4^iii^	89.89 (8)	O2—C7—O1	128.0 (2)
O1—Cu1—O4^iii^	88.48 (8)	O2—C7—C1	116.9 (2)
O3^ii^—Cu1—O4^iii^	167.51 (8)	O1—C7—C1	115.0 (2)
O2^i^—Cu1—O5	98.84 (8)	O3—C8—O4	126.9 (2)
O1—Cu1—O5	93.64 (8)	O3—C8—C3	117.2 (2)
O3^ii^—Cu1—O5	98.51 (8)	O4—C8—C3	115.9 (2)
O4^iii^—Cu1—O5	93.91 (8)	O5—C9—H9A	109.5
O2^i^—Cu1—Cu1^i^	84.70 (5)	O5—C9—H9B	109.5
O1—Cu1—Cu1^i^	82.79 (6)	H9A—C9—H9B	109.5
O3^ii^—Cu1—Cu1^i^	85.21 (6)	O5—C9—H9C	109.5
O4^iii^—Cu1—Cu1^i^	82.31 (5)	H9A—C9—H9C	109.5
O5—Cu1—Cu1^i^	174.85 (7)	H9B—C9—H9C	109.5
C6—C1—C2	117.1 (2)	O6—C10—H10A	109.5
C6—C1—C7	121.9 (2)	O6—C10—H10B	109.5
C2—C1—C7	120.9 (2)	H10A—C10—H10B	109.5
F1—C2—C1	117.0 (2)	O6—C10—H10C	109.5
F1—C2—C3	118.9 (2)	H10A—C10—H10C	109.5
C1—C2—C3	124.0 (2)	H10B—C10—H10C	109.5
C4—C3—C2	116.4 (2)	C7—O1—Cu1	123.16 (17)
C4—C3—C8	121.5 (2)	C7—O2—Cu1^i^	121.24 (15)
C2—C3—C8	122.1 (2)	C8—O3—Cu1^iv^	121.44 (17)
F2—C4—C5	117.8 (2)	C8—O4—Cu1^v^	124.10 (16)
F2—C4—C3	120.6 (2)	C9—O5—Cu1	126.70 (19)
C5—C4—C3	121.5 (2)	C9—O5—H5	108 (3)
F3—C5—C6	120.6 (3)	Cu1—O5—H5	120 (3)
F3—C5—C4	119.8 (3)	C10—O6—H6	109.5
C6—C5—C4	119.6 (3)		

Symmetry codes: (i) −*x*+2, −*y*+2, −*z*+1; (ii) *x*+1/2, −*y*+5/2, *z*+1/2; (iii) −*x*+3/2, *y*−1/2, −*z*+1/2; (iv) *x*−1/2, −*y*+5/2, *z*−1/2; (v) −*x*+3/2, *y*+1/2, −*z*+1/2.

### Hydrogen-bond geometry (Å, º)

**Table:**

*D*—H···*A*	*D*—H	H···*A*	*D*···*A*	*D*—H···*A*
O5—H5···O6^vi^	0.84 (1)	1.80 (1)	2.637 (3)	173 (4)
O6—H6···O4^vii^	0.82	2.02	2.828 (3)	169

Symmetry codes: (vi) *x*+1/2, −*y*+3/2, *z*+1/2; (vii) *x*−1, *y*−1, *z*.
